# Antibacterial Nanocellulose-TiO_2_/Polyester Fabric for the Recyclable Photocatalytic Degradation of Dyes

**DOI:** 10.3390/polym15224376

**Published:** 2023-11-10

**Authors:** Jiacheng Tan, Hangjun Deng, Fangfang Lu, Wei Chen, Xiuping Su, Hairong Wang

**Affiliations:** 1 Key Laboratory of Functional Fibers and Intelligent Textiles, Yuanpei College, Shaoxing University, Shaoxing 312000, China; 13587930849@163.com (J.T.); 13758210233@163.com (H.D.); 2 Zhoushan Institute of Calibration and Testing for Quality and Technology Supervision, Zhoushan 316000, China; 17826854282@163.com; 3 College of Textile Science and Engineering, Zhejiang Sci-Tech University, Hangzhou 310018, China; cw@zstu.edu.cn

**Keywords:** TiO_2_, polyester, nanocellulose, photocatalytic degradation, antibacterial property

## Abstract

In this paper, we report an antibacterial, recyclable nanocellulose–titanium dioxide/polyester nonwoven fabric (NC-TiO_2_/PET) composite for the highly efficient photocatalytic degradation of dyes. The NC-TiO_2_ was loaded onto the surface of flexible PET nonwoven fabric through a simple swelling and dipping method. The NC-TiO_2_ in the particle size range of ~10 nm were uniformly attached to the surface of the PET fibers. The NC-TiO_2_/PET composite has the ability to achieve the stable photocatalytic degradation of dyes and presents antibacterial properties. The degradation rates to methylene blue (MB) and acid red (AR) of the NC-TiO_2_/PET composite reached 90.02% and 91.14%, respectively, and the inhibition rate of *Escherichia coli* was >95%. After several rounds of cyclic testing, the photocatalytic performance, antibacterial performance, and mechanical stability of the NC-TiO_2_/PET composite remained robust.

## 1. Introduction

Despite the clean, non-toxic tap water consumed by most urban dwellers in modern societies, water pollution still poses a threat to less developed regions [1,2]. Also, the coronavirus pneumonia that has been raging over the past few years reminds us, once again, of the threat that microorganisms and viruses pose to human life and health [3,4]. Dye-containing wastewater can destroy the ecological balance and also cause cancer if consumed by organisms (including humans) for long periods of time, even at low concentrations [5,6,7]. Therefore, researchers have invested significant efforts in studying wastewater treatment materials [8].

Titanium dioxide (TiO_2_), a nontoxic, cheap, stable, and excellent photocatalyst, shows its excellent treatment ability for various types of water pollutants [9,10]. However, the large-scale application of nanoparticles still poses problems, such as their easy aggregation and difficulties in their separation from water treatment systems. Recently, the doping of TiO_2_ with nonmetals and metals or its coupling with other semiconductors have been studied to improve its photocatalytic performance. Li et al. [11], using the co-graft polymerization of 2-hydroxyethyl acrylate (HEA) with TiO_2_ nanoparticles on cotton fabrics via γ-ray irradiation, prepared a photocatalytic composite highly efficient against organic pollutants. Alhalili et al. [12] removed dithioterethiol (DTT) from water using membranes of cellulose acetate (AC) and AC-doped ZnO/TiO_2_ nanoparticles.

Other scientists are embedding nanomaterials into fibers to advance their stability. Wang et al. [13] used a layer-by-layer (LBL) self-assembly strategy to load TiO_2_/g-C_3_N_4_ on cotton fabrics in order to obtain an efficient, stable, and reusable visible light catalytic functional material. However, to further improve the stability of organic–inorganic composites, we found that it is crucial to select efficient organic and inorganic linkers. Nanocellulose (NC) is an ideal environmentally friendly material that is sustainable and inexpensive [14]. NC can be extracted from plants, bacteria, and marine shellfish, meaning that it is degradable, flexible, hydrophilic, and rich in surface functional groups [15]. NC can act as an interfacial “bridge” between polymers and inorganic nanomaterials, making it ideal for the interfacial chemistry of composite materials [16,17,18]. Xue et al. [19] report a limited growth strategy mediated by the self-assembly of cellulose nanocrystal (CNC) to prepare anatase TiO_2_ nano-catalyst with dominantly exposed (001) reactive facets and hierarchical pore structure for high photocatalytic activity.

Herein, this work aims to provide a simple, low-cost, and recyclable material for the treatment of water bodies. Its recycling and reusability can be facilitated through the use of a polyester nonwoven substrate. The TiO_2_ was stably loaded onto the polyester nonwoven fabric using a simple swelling and dipping method, and the NC was introduced as a “bridge” to facilitate the dispersion of the nanomaterials and make them firmly bonded to each other. Finally, stable NC-TiO_2_/PET composites were synthesized to treat organic pollutants and eliminate bacteria in environmental water bodies.

## 2. Experimental Section

### 2.1. Materials

Polyester nonwovens (PET) with a gram weight of 135 g m^−2^ were purchased from Texas Hongrui Geomaterials Co., Ltd. (Shandong, China). Cellulose, titanium dioxide nanoparticles, methylene blue (MB), acid red (AR), sodium hydroxide (NaOH), and cetyltrimethylammonium bromide (CTAB) were purchased from Aladdin Chemistry Co., Ltd. (Shanghai, China).

### 2.2. Alkali Reduction Pretreatment of Fabric and Preparation of NC

A certain amount of clean polyester nonwoven fabric was washed and immersed in an alkali reduction treatment solution (5 g L^−1^ sodium hydroxide and 1 g L^−1^ CTAB) with a bath ratio of 1:20. Then, it was stirred at 95 °C for 30 min, washed, and dried. The PET fabrics used in this paper were subjected to the alkali reduction treatment. The NC was obtained by dissolving cellulose powder in an alkaline urea solution (7 wt% of NaOH and 12 wt% of urea). After the reaction, the NC solutions were filtered and freeze-dried at −70 °C for 72 h to obtain the dry NC.

### 2.3. Preparation of NC-TiO_2_/PET

A total of 0.2 wt% of NC and 0.2 wt%–1.0 wt% of nano-titanium dioxide (TiO_2_) were ultrasonically dispersed for 30 min to obtain the NC-TiO_2_ suspension. Then, the pretreated PET fabric was dipped into the NC-TiO_2_ suspension for 20 min and put under 0.3 MPa roll pressure for one dip and one tie. Then, it was baked in a shaping dryer at 90 °C for 5 min, and the procedure for impregnating the rolls and drying them was repeated twice. Finally, the samples were cleaned and then transferred into an 80 °C vacuum-oven and baked for 40 min to obtain the NC-TiO_2_/PET samples. We also prepared a group of samples without the NC, named TiO_2_-PET.

### 2.4. Characterization

The micromorphology and elemental composition of the materials were characterized using a field emission scanning electron microscope (FE-SEM, Sigma 300, ZEISS, Oberkochen, Germany) with energy-dispersive X-ray spectroscopy (EDS). The TiO_2_ content on the fabric was quantified using a atomic absorption spectrometer (AAS, Solaar M6, Thermo, Waltham, MA, USA) with an air-acetylene flame. Separate hollow cathode lamps radiating at wavelengths of (Ti) 248.3 nm were used to determine the amount of TiO_2_. The material phases of dried NC-TiO_2_/PET fabric and PET fabric surface structures were analyzed using XRD. The crystal phases in the samples were analyzed using X-ray diffractometry (XRD, D8, Bruker, Frankfurt, Germany). Fourier-transform infrared (FTIR) spectrometry (FTIR, Nicolet IS50, Thermo, Waltham, MA, USA), equipped with an ATR accessory, was used to examine the functional groups of the samples. All the spectra were recorded in the wavenumber range from 4000 to 450 cm^−1^, with a 4 cm^−1^ resolution. An X-ray photoelectron spectroscopy (XPS, K-Alpha Thermo, Waltham, MA, USA) was performed using an electronic spectrometer. The mechanical strength of the samples was tested through a universal testing machine (Instron 5943, Norfolk, MA, USA).

### 2.5. Photocatalytic Performance

The PET and NC-TiO_2_/PET materials were cut into sizes of 2 × 2 cm. First, the mock-contaminants MB (20 mg L^−1^) and AR (20 mg L^−1^) were prepared, and then 150 mL of these solutions was poured into clean Petri dishes. The NC-TiO_2_/PET and PET nonwoven fabrics were added to the Petri dishes and left in a dark room for 2 h to reach adsorption equilibrium. After reaching the adsorption equilibrium, the dishes were transferred to a 16 W UV lamp (λ ≈ 280 nm) for light irradiation, and the vertical irradiation distance between the dishes and the UV lamp was adjusted to a uniform 20 cm. Color changes of the fabric’s surface and of the contaminant solution in the dishes were observed 30 min, 60 min, 120 min, and 180 min after light irradiation had begun. Changes in the dye solutions’ concentration were measured under a UV spectrophotometer and recorded using digital photography. The absorbance of the MB and AR dye solutions were tested at 664 nm and 532 nm. All the photocatalytic degradation experiments were performed three times, and the average value was taken. The degradation rate of the dyes was analyzed using the UV absorption spectrum. The degradation rates (%) of the MB and AR solutions were calculated as follows:

(1) $$\mathrm{Degradation}\;\mathrm{rate}\left(\%\right)=\frac{C_{0}-C}{C_{0}}\;100\%$$

where C is the dye (MB and AR)’s concentration (mg L^−1^) at time t (min), and C_0_ is the initial dye concentration (mg L^−1^). All the experiments of photocatalytic degradation of the dyes were carried out three times, and the average value was taken.

### 2.6. Antibacterial Performance

The Gram-negative bacterium *Escherichia coli* (*E. coli*, ATCC 25922) was selected to test the antibacterial performance of the NC-TiO_2_/PET. The procedure was carried out on a clean bench, and all the materials used in the antimicrobial activity test were sterilized in an autoclave. The samples were evaluated for their antibacterial performance using the qualitative agar diffusion method and the quantitative mean colony-forming unit method [20]. The antimicrobial rates of the samples were calculated as follows:

(2) $$\mathrm{Antimicrobial}\;\mathrm{Rates}\left(\%\right)=\frac{N_{0}-N_{1}}{N_{\begin{array}{l} 0 \end{array}}}\;100\%$$

where *N*_1_ is the average number of *E. coli* in the liquid medium in which the NC-TiO_2_/PET was immersed, and *N*_0_ is the average number of *E. coli* in a pure medium with normal bacterial growth.

## 3. Results and Discussion

### 3.1. Microscopic Surface Structures

**Figure 1:**
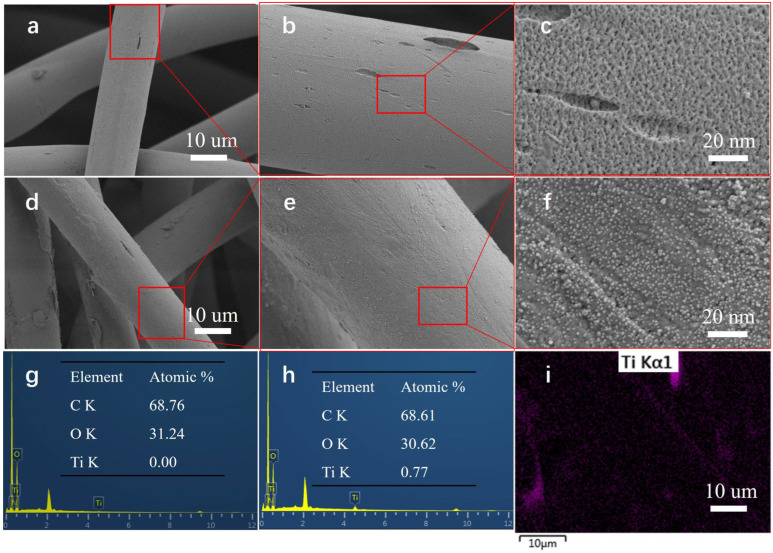
Microstructure and elemental compositions of PET and NC-TiO_2_/PET. (**a**–**c**) FE-SEM images of PET; (**d**–**f**) FE-SEM images of NC-TiO_2_/PET; (**g**,**h**) EDS of PET and NC-TiO_2_/PET, respectively; and (**i**) EDS mapping of NC-TiO_2_/PET.

The microscopic surface structures of the PET and NC-TiO_2_/PET were observed using FE-SEM. From Figure 1a–c, it can be seen that the surface structure of the pretreated polyester fiber was rough and that the fiber’s diameter was about 10–20 μm. Figure 1d–f shows the magnified images of the NC-TiO_2_/PET. The high-resolution FESEM image in Figure 1f shows that the surface of the NC-TiO_2_/PET had an even distribution of nanoparticles (NPs) on the individual fibers and that the average diameter of these NPs was 10 nm. Compared to the pure PET fabric, the atomic percentage of Ti on the NC-TiO_2_/PET surface reported was about 0.77%, according to the EDS results in Figure 1g,h. Furthermore, the EDS mapping in Figure 1i indicates that the TiO_2_ was densely and homogeneously distributed on the surface of the PET fabric.

### 3.2. Chemical Characterizations

**Figure 2:**
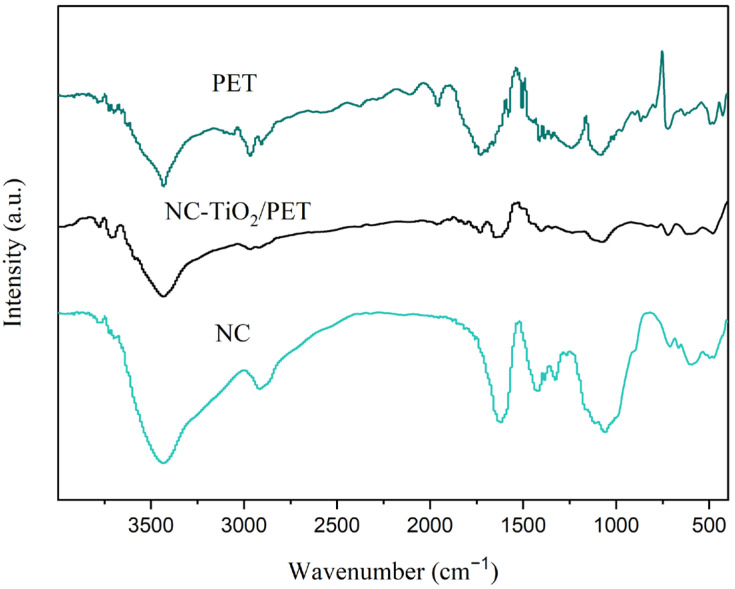
FTIR spectra of PET, NC-TiO_2_/PET, and NC.

The chemical characterization of the PET, NC, PET-TiO_2_, and NC-TiO_2_/PET were performed using FTIR spectroscopy. In Figure 2, it can be seen that the characteristic peak of the -C=O in the PET was observed at 1700 cm^−1^. The peaks at 1090 and 1240 cm^−1^ were the vibrations of the -C-O groups in the PET. The characteristic peaks at 1015 cm^−1^ and 720 cm^−1^ corresponded to the -C-H of the PET. Compared to the PET, the characteristic peak at 2899 cm^−1^ in the NC-TiO_2_/PET was the typical band for the cellulose nanomaterial molecules, and the strong characteristic peaks below 700 cm^−1^ were due to the bending of the -Ti-O bonds in the NC-TiO_2_/PET [21]. In addition, the characteristic peaks of the cellulose nanofibers were clearly visible in the broad bands’ 3600–3000 cm^−1^ region and were caused by telescopic vibrations within and between the O-H bonds. The large absorption in the 1200–860 cm^−1^ spectral region was caused by the vibrations of the glucose rings of C-H, C-O, and C-O-C of the NC-TiO_2_/PET. The FTIR of the NC-TiO_2_/PET showed intense absorption peaks associated with O-H stretching at 3430 cm^−1^ and O-H bending at 1630 cm^−1^, which were similar to those observed in the NC, which were shifted to lower wavenumbers. The observed shift was related to the involvement of the -OH groups in the formation of strong, intermolecular hydrogen-bonding interactions between the hydroxyl groups in the cellulose chains and the Ti-OH groups in the TiO_2_ nanoparticles. Interestingly, since -OH promotes the formation of -OH radicals in the photocatalytic process, the increase of surface groups’ -OH may positively affect the photodegradation activity of the NC-TiO_2_ nanocomposites [22].

**Figure 3:**
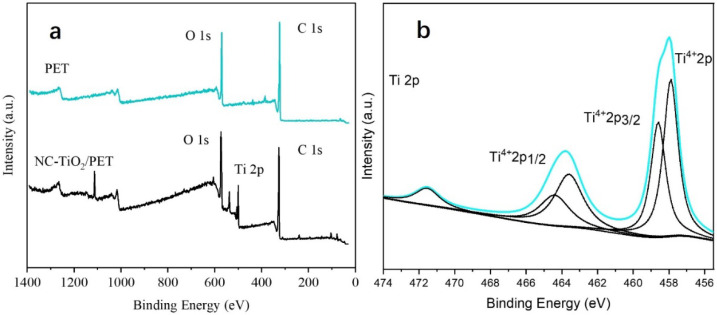
XPS spectra of PET and NC-TiO_2_/PET. (**a**) XPS survey spectra of PET and NC-TiO_2_/PET. (**b**) High-resolution XPS 2p spectra of NC-TiO_2_/PET.

Figure 3a shows the XPS survey spectra of the PET and NC-TiO_2_/PET, which show that the PET contained only C and O, while the NC-TiO_2_/PET also contained Ti. Figure 3b shows the Ti 2p spectra, where the peaks of 2p_1/2_ and 2p_3/2_ appeared at 464.3 eV and 458.5 eV, respectively, indicating the presence of Ti^4+^ (TiO_2_) [23].

### 3.3. Crystal Phase Structure Analysis

**Figure 4:**
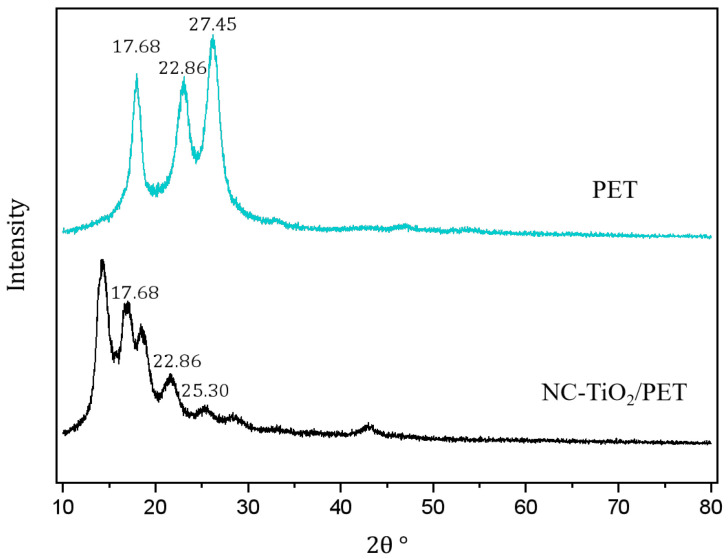
XRD patterns of PET and NC-TiO_2_/PET.

The XRD pattern of the PET showed a characteristic diffraction peak of polyester fiber at 2θ = 17.68° and another peak near 22.86° (Figure 4). The XRD pattern of the NC-TiO_2_/PET showed a characteristic diffraction peak of TiO_2_ at 27.45°, in addition to the characteristic diffraction peak of the PET. The characteristic diffraction peak of the (110) and (111) crystal planes of the TiO_2_ appeared at 27.45° and 42°, respectively, which are consistent with results in the previous literature [24]. This implies that the surface of the polyester fiber was successfully loaded with anatase-phase TiO_2_ nanoparticles.

### 3.4. Performance of Photocatalytic Degradation

TiO_2_ nanomaterials are widely used because of their excellent photocatalytic activity, and the use of UV light for removing organic dyes has become an area of great interest in the water treatment field [25]. Most previous photocatalytic materials for treating dye wastewater have been powder materials, which are difficult to recycle and reuse in practical applications. Therefore, in this study, PET fabric was used as a flexible substrate, onto which TiO_2_ nanomaterials were loaded to facilitate recycling and avoid secondary pollution.

The effects of the composition ratio on the catalytic degradation properties of the NC-TiO_2_/PET materials are shown in Table 1. The ratios of NC to TiO_2_ in the preparation of the composite materials were determined after a preliminary experiment optimization. The degradation rate of the MB was increased from 65.07% to 90.02%, when the mass ratio of the NC/TiO_2_ was increased from 0.2 wt%/0.4 wt% to 0.2 wt%/0.8 wt%. However, when the ratio reached 0.2 wt%/1.0 wt% of NC/TiO_2_, the catalytic efficiency began to decrease, as the nanoparticles on the surface of the sample tended to aggregate. The sample which was formed using 0.2 wt%/0.8 wt% of NC/TiO_2_ exhibited an excellent catalytic degradation ability.

**Figure 5:**
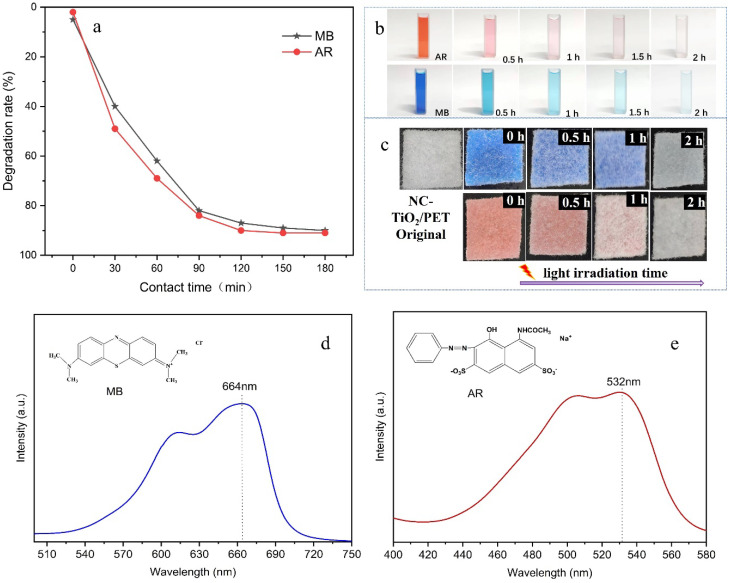
Degradation rate curves of the NC-TiO_2_/PET for the MB and AR dyes at different times (**a**); photos of the dye solution and the NC-TiO_2_/PET materials at different photocatalytic light irradiation times, (**b**,**c**); and the UV-vis spectra and chemical formulas of the dyes MB (**d**) and AR (**e**).

To investigate the photocatalytic performance of the NC-TiO_2_/PET materials, cationic methylene blue (MB) and anionic acid red (AR) dyes were used as model contaminants to determine the photocatalytic decomposition activity of the materials against the dyes under UV light. To track changes in their concentration, the absorbance of the MB and AR dye solutions were tested at 664 nm and 532 nm, respectively, from the UV-vis absorption peak (Figure 5a,b). Samples of 2 × 2 cm^2^ of fabric were immersed in two Petri dishes with certain concentrations of dyes, and the medium was placed in a dark room for 2 h to reach the absorption–desorption equilibrium. Subsequently, the photocatalytic reaction was performed by exposing the solution to a UV lamp. Six reaction times were set to degrade the MB and AR dye solutions using the NC-TiO_2_/PET composite; these were 0, 30, 60, 90, 120, and 180 min, respectively. The concentration of the dye solution sampled from the Petri dishes at certain irradiation times was measured to monitor the photocatalytic reaction, and the results are shown in Figure 5. The chromaticity of the solution began to fade at 0.5 h of illumination, and the concentration of the MB and AR dye solutions decreased by 40.22% and 49.19% (Figure 5d,e), respectively. The color of the solutions continued to fade after 60 min of illumination, and the chromaticity of both MB and AR decreased significantly at 180 min of illumination. The degradation rates of the MB and AR solutions reached 90.02% and 91.14%, respectively. We also observed a significant change in the NC-TiO_2_/PET fabric, as shown in Figure 5c. The color of the surface of the dye-contaminated nonwoven fabric was slightly faded after 30 min of illumination, greatly faded at 60 min, and almost completely faded after 2 h of illumination. As a control, we also used pure PET materials for the photocatalytic degradation of pollutants. The results showed that the dye degradation rate was 6.47% after 3 h of illumination. It could be judged that the NC-TiO_2_/PET fabric has an efficient photocatalytic degradation ability against organic pollutants.

During UV irradiation of the NC-TiO_2_/PET, a series of physicochemical reactions occurred in the solution and on the surface of the NC-TiO_2_/PET, including the following: the TiO_2_ absorbed energy (hv) to electrons (*e*^−^) and generated holes (h^+^), and these *e*^−^ and h^+^ reacted further with oxygen and water to produce reactive oxygen species (ROS) such as superoxide radicals (·O_2_^−^) and hydroxyl radicals (·OH). The photocatalytic degradation mechanism can be deduced as follows [23]:

NC-TiO_2_/PET + hv → NC-TiO_2_/PET^+^ + *e^−^* (3)

*e*^−^ + O_2_ → ·O_2_*^−^* (4)

NC-TiO_2_/PET^+^ → NC-TiO_2_/PET + h^+^ (5)

h^+^ + H_2_O → ·OH + h^+^ (6)

ROS + MB → CO_2_ + H_2_O (7)

Firstly, the ·OH radical attacks the -N = bond and the -S = bond of the MB to interrupt the MB molecule. In the second step, the formed ROS degrades the dye molecules. The MB split product is oxidized to diphenol, phenol, etc., because diphenol and phenol have an electron donor -OH present on the benzene ring, which is beneficial to ·OH electrical attacks, especially in the presence of -OH on the para and ortho positions. The diphenol can be quickly oxidized to benzoquinone, and the phenol can be oxidized to catechol, resorcinol, hydroquinone, etc. Thirdly, the intermediate product of the aromatic ring is further oxidized by ·OH into a short-chain carboxylic acid such as oxalic acid, fumaric acid, formic acid, etc., and these short-chain carboxylic acids can be rapidly degraded into non-toxic and harmless water and carbon dioxide [13]. The final product of the photocatalytic decomposition of acid red using TiO_2_ is H_2_O and CO_2_ [26].

### 3.5. Recycling Performance and Stability

**Figure 6:**
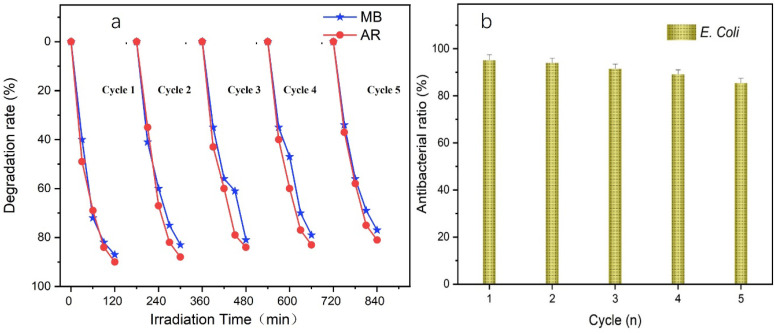
(**a**) Photocatalytic cyclic degradation of MB and AR using NC-TiO_2_/PET; (**b**) circulating antibacterial rate of NC-TiO_2_/PET against bacteria.

Flexible fabrics are easily reusable, and the photocatalytic degradation performance of the NC-TiO_2_/PET samples in this study was tested over five reuse cycles. We performed the cycles by simply rinsing the samples with distilled water. Figure 6a shows that the photocatalytic degradation rate of the NC-TiO_2_/PET was maintained at 81.08% for the MB and at 77.25% for the AR after five cycles. For the cycling experiments of the TiO_2_-PET, the first degradation rate of the MB was 89.20% at 2 h, 56.17% for the 2nd cycle, and decreased to 35.09% for the 3rd cycle. The TiO_2_-PET without the NC had a poor cycling stability relative to the NC-TiO_2_/PET. These results verify that the NC acted as a bridge between the nanoparticles and the fibers to stabilize the connection between the two [27].

To further investigate the stability of the material during use, we examined changes in its mechanical strength before and after cycling, and its tensile fracture was tested using a universal mechanical tester. The results indicated that the strength and durability of the material were good after recycling.

### 3.6. Antibacterial Properties and Stability

Generally, nanoscale TiO_2_ has strong antibacterial properties, so the samples were tested for their antibacterial properties using Gram-negative bacteria *E. coli* as a representative. The antibacterial properties of the materials were determined using plate colony counts. Table 2 shows that the CFU (colony forming units) counts of the *E. coli* colonies on the media decreased to 142 after the NC-TiO_2_/PET was immersed in the bacterial test tubes for 30 min. The CFU of the blank, control PET fabric remained at 602. The antibacterial rates of the NC-TiO_2_/PET against *E. coli* reached 95.12%, indicating that the composite had a strong antibacterial activity. Normally, antibacterial hydrogen peroxide (H_2_O_2_) is produced on the surface of titanium dioxide and binds to bacterial cell membranes, affecting their permeability and respiratory function, and, eventually, leading to bacterial death [28]. In addition, experiments were conducted to verify the antibacterial ability of the NC-TiO_2_/PET and the stability of the material for five cycles (Figure 6b). After five cycles, the NC-TiO_2_/PET showed an excellent stability against *E. coli* by maintaining an antimicrobial rate of 85.24% and CFU counts of 201. This study showed that the NC-TiO_2_/PET composite has a stable and long-lasting antimicrobial performance for use in aqueous environments.

## 4. Conclusions

In this study, excellent antibacterial PET composite materials with recyclable photocatalytic degradation properties were achieved by incorporating nano-TiO_2_ through a simple swelling and dipping method and fixing them with NC. The porous and loose structure of the fabrics provides a high adsorption ability against pollutants and impels the active species to quickly capture pollutants in order to degrade them. As a result, the NC-TiO_2_/PET composite presents an efficient photocatalytic performance for both cationic dye MB and cationic dye AR liquid pollutants’ decomposition. Under UV irradiation, the composite showed both decomposition and removal effects on methylene blue (90.02%) and acid red (91.14%), and the composite’s inhibition rate against *E. coli* was 95.12%. Finally, the photocatalytic degradation, antibacterial experiments, and AAS test results showed that the composite material exhibits good stability during recycling.

## Figures and Tables

**Table 1:** The degradation rate (MB) of samples synthesized with various mass ratios of NC/TiO_2_ for 3 h.

Sample	NC/TiO_2_	Degradation Rate (%)
PET	0	6.47
TiO_2_/PET	0 wt%/0.8 wt%	89.79
NC-TiO_2_/PET-0.4	0.2 wt%/0.4 wt%	65.07
NC-TiO_2_/PET-0.6	0.2 wt%/0.6 wt%	79.37
NC-TiO_2_/PET	0.2 wt%/0.8 wt%	90.02
NC-TiO_2_/PET-1.0	0.2 wt%/1.0 wt%	79.71

**Table 2:** Results of AAS, mechanical properties, and antibacterial ratios of the samples.

Sample	TiO_2_ per NC-TiO_2_/PET (mg g^−1^)	Breaking Strength (N)	Antibacterial Properties against *E. coli* (CFU mL^−1^)
PET	--	470.21 ± 3.59	602
NC-TiO_2_/PET	10.11 ± 0.78	468.54 ± 3.44	142
NC-TiO_2_/PET after Fifth cycle	9.29 ± 0.86	448.17 ± 3.57	201

## Data Availability

There are legitimate reasons to request data from the corresponding author.
